# Supplementary material for: Projective diffeomorphic mapping of molecular digital pathology with tissue MRI
Source: Commun Eng. 2022 Dec 13;1:44. doi: 10.1038/s44172-022-00044-1 (PMC10243734; doi:10.1038/s44172-022-00044-1)
Supplement: Supplementary file 2 — Supplementary Information [file 44172_2022_44_MOESM2_ESM.pdf]

Supplementary Information

Supplementary Note 1

Two-dimensional (2D) positron emission tomography (PET) introduces point-spreads $p_n(y, dx), y \in R^2, dx \subset R^2$ for reconstruction which are supported over planes $Y \subset R^2$ with uncertainty perpendicular to the line of flight but as well a second measurement the time-of-flight of the annihilating protons to the detectors [1]. Generally the point-spreads are modelled as cigar shaped two-dimensional Gaussians in the plane oriented by N angles $\theta_n, n = 1, \ldots, N$, with high fidelity systems having $N > 96$, with standard deviation of uncertainty significantly larger along the lines of flight than perpendicular to them.

Classical parallel beam projection tomography reconstructs image planes $Y \subset R^2$ via the Radon transform generated from sinograms indexed over a single space dimension $Z \subset R^1$ arising from idealized line integrals [2]. We define the set of oriented lines in R^2 parametetrized by their angles (θ) and offsets from the origin (z), $L_\theta(z) = \{(y^{(1)}, y^{(2)})\} \subset R^2$ with

$$\begin{aligned} y^{(1)} &= \bar{z}\sin(\theta) + z\cos(\theta) \\ y^{(2)} &= -\bar{z}\cos(\theta) + z\sin(\theta) \end{aligned}, \tag{S1}$$

for indexing variable $\bar{z} \in R^1$. To satisfy the basic sampling theorems for tomographic reconstruction [3], N sampling angles $\theta_n, n = 1, \ldots, N$, akin to the sections in histology, are selected determined by the resolution required for the reconstruction. The Radon transform is typically written in functional notation as an integral along one dimension, with Lebesgue measure as:

$$R_\theta I(z) = \int_R I(\bar{z}\sin(\theta) + z\cos(\theta), -\bar{z}\cos(\theta) + z\sin(\theta))d\bar{z}. \tag{S2}$$

It can also be modeled as the integral over the line parameterized by (θ, z) as defined above:

$$R_\theta I(z) = \int_{L_\theta(z)} I(y)dy, \quad y \in R^2. \tag{S3}$$

In the notation introduced in the "Projective LDDMM" subsection in the Results, we extend this to an integral over all of R^2, using Dirac delta measures that assign nonzero measure only to lines in this same set:

$$R_\theta I(z) = \int_{R^2} I(y)\delta_{L_\theta(z)}(dy), \quad y \in R^2. \tag{S4}$$

The line integral indexed by (θ, z) is modeled as a single projection $P_n I(z), z \in R$ indexed by a set of $n = 1, ..., N$ and is given as in (4b, main text), with point spread, $p_n(z, dy) = \int_{L_{\theta_n}(z)} ds\delta_s(dy)$. We show below its equivalence to

the line integral as defined in above (S3).

$$\begin{aligned}
P_n I(z) &= \int_Y p_n(z, dy) I(y) \\
&= \int_Y \int_{L_{\theta_n}(z)} ds \delta_s(dy) y I(y) \\
&= \int_{L_{\theta_n}(z)} \int_Y \delta_s(dy) I(y) ds, \quad \text{by Fubini's Theorem} \\
&= \int_{L_{\theta_n}(z)} I(s) ds, \quad s \in R^2
\end{aligned}$$

Supplementary Note 2

Similar to other groups [4], we evaluated accuracy of alignment between 2D histology and 3D MRI by looking at sets of discrete points (pixels) labeled in 2D versus corresponding voxels labeled in 3D and subsequently deformed to 2D (see Figure 5). Here, our sets of points were the sets of pixels (voxels) within a particular MTL subregion as delineated on 2D histology images and 3D MRI (see the "Segmentations of MTL Subregions" subsection of the Methods). We restricted our attention to particular subregions of interest (CA fields, dentate gyrus, and subiculum), and measured accuracy by Dice Score and 95th Percentile Hausdorff distance for each region on each slice of one brain. Average overlap scores were 0.65, 0.75, 0.72, 0.84 for subiculum, CA fields, dentate gyrus and whole hippocampus, respectively while average 95th percentile Hausdorff distance was 1.76 mm, 1.43 mm, 1.21 mm, and 1.69 mm for subiculum, CA fields, dentate gyrus, and whole hippocampus, respectively.

Supplementary Note 3

The Scattering Transform, by Mallat and Bruna [5, 6], defines a cascade of alternating non-linear and non-commuting operators that generate from an image, $J_n(\cdot) \in L^2(R^d)$, a set of "filtered images" $(S^1_{J_n}(\cdot), S^2_{J_n}(\cdot), \dots)$ that capture textural information in the original image. Each "filtered image" is generated by a scattering propagator, U that takes the original image J_n down a particular path, p, of alternating convolutions with wavelets (localized waveforms) and modulus operations. The path, p, is defined by a set of parameters $\lambda \in 2^{\mathbb{Z}}$ that scale a mother wavelet, ζ, so as to capture lower and lower frequency information.

$$\begin{aligned}
p &= (\lambda_1, \lambda_2, \cdots, \lambda_\ell) \\
U[p] J_n &= |||J_n \star \zeta_{\lambda_1}| \star \zeta_{\lambda_2} \cdots | \star \zeta_{\lambda_s}|
\end{aligned} \tag{S5}$$

In our setting, we compute a subsampled Scattering Transform, $\mathcal{S}$, of each of our histology images, using an algorithm similar to the "Filterbank" algorithm [7, 8] in which images are downsampled in parallel with scattering. The path dependent propagator corresponding to $\mathcal{S}$ is $U_s[\cdot]$:

$$U_s[P] : J_n(\cdot) \mapsto (S^1_{J_n}(\cdot), \ldots, S^{48}_{J_n}(\cdot)) \tag{S6}$$

$$J_n : R^2 \to R^q, \quad S^s_{J_n} : R^2 \to R, \quad s = 1, \ldots, 48$$

$$P = (p_1, p_2, \cdots, p_{16}), \ell \in \{1, 2\}, \lambda_i \in \{2^0, 2^1, 2^2, 2^3, 2^4, 2^5\} \ .$$

We use 16 paths, $p_i \in P$ of length 1 or 2 and a high pass Gaussian filter, $(1 - \frac{1}{2\pi\lambda_i}\exp(-\frac{(x^2+y^2)}{2\lambda_i^2}))$, with width dilated according to λ_i, in place of a traditional wavelet to achieve a representation both translation and rotation invariant in addition to Lipschitz continuous to small deformations. Each of the R,G,B channels of histology images are propagated independently along the same paths. Histology images are downsampled by a factor of 32 to reach the approximate resolution of MRI. Together, the subsampling and scattering of each channel yield a total of 48 scattering coefficients for each pixel in the downsampled histology image, or 48 "filtered images" per target histology image.

Supplementary Note 4

For computing and comparing NFT density measures across brain samples, we rigidly aligned our brain sample to the reference brain in the Mai Paxinos Atlas [9]. Surface renderings of the hippocampus, amygdala, and ERC in the Mai atlas were constructed from coronal images at every 1.3 mm using restricted Deulaney triangulation [10]. We used a manual alignment tool, created in-house, to select optimal alignments between these surface renderings and those of our brain sample, constructed from manual segmentations on 3D MRI. Alignments were qualitatively confirmed by comparison of landmarks on corresponding Mai surfaces and transformed surfaces of our brain sample (e.g. anterior and posterior poles of hippocampus and folds in hippocampal head). These appeared within 3-4 mm of each other. Given that coronal sections in the Mai atlas in this area are approximately 1.3mm apart, we expect accuracy of reported Mai coordinates on the order of 2-4mm.

Distributions of NFT density were computed in the coordinates of the Mai atlas, according to choice of $\pi(x, x')$ governing physical spatial spread, and $\gamma(\cdot)$ governing smoothing over conditional feature distributions (17, main text). In all cases, total mass (2D cross-sectional tissue area of histological images) and total number of NFTs were conserved. To achieve this, initial NFT feature values (counts per MTL subregion) were reformulated following physical transformation (14a, main text) as counts of NFTs per MTL subregion

per weight of particle (i.e. total cross-sectional area):

$$f_i^j \mapsto \begin{cases} \frac{f_i^j}{w_i} & \text{for } 1 \leq j \leq \ell \\ f_i^j & \text{for } \ell + 1 \leq j \leq 2\ell \end{cases} \tag{S7}$$

We highlight three different modes of resampling. Volumetric resampling (e.g. at mm resolution) was computed with a 3D isotropic Gaussian kernel with width, σ, and with new particles in a regular lattice, $x' \in X'$.

$$\pi(x, x') := \frac{1}{\zeta} \exp\left(- \frac{\|x - x'\|_2^2}{2\sigma^2} \right) \tag{S8a}$$

$$\zeta := \sum_{x' \in X'} \pi(x, x') = 1 \tag{S8b}$$

Resampling over 2D manifolds (e.g. the surface of CA1 or Subiculum) was computed using a nearest neighbor kernel, assigning all weight (tissue area) and NFTs from a particle at the fine scale to a single particle on the 2D manifold (e.g. vertex of a triangular mesh).

$$\pi(x, x') := \begin{cases} 1 & \text{if } x' = \arg\min_{X'} \|x - x'\|_2^2 \\ 0 & \text{otherwise} \end{cases} \tag{S9}$$

Finally, resampling to a regular 1D lattice (e.g. the rostral-caudal axis of the human brain) was computed using an anisotropic Gaussian kernel to spread particle mass widely in two dimensions and narrowly in the third, with dimensions treated independently.

$$\begin{aligned} \pi(x, x') &:= \frac{1}{\zeta} \exp\left(- \frac{(x - x')^T \Sigma^{-1} (x - x')}{2} \right) \\ \zeta &:= \sum_{x \in X'} \pi(x, x') = 1 \\ \Sigma &:= \begin{bmatrix} \sigma_a^2 & 0 & 0 \\ 0 & \sigma_a^2 & 0 \\ 0 & 0 & \sigma_b^2 \end{bmatrix}, \quad \sigma_a >> \sigma_b \end{aligned} \tag{S10}$$

In each case, feature reduction occurred via computation of expected first moments, as described in the "Particle Representation of Histological Data" subsection in the Methods.

Supplementary Note 5

The variational solution to (14) is given by:

$$(Id - k\Delta)\hat{g} = g \tag{S11}$$

where Δ is a Laplacian operator. Here, we take Δ as the Laplace Beltrami operator and compute an eigenbasis ($\mathcal{B} = \{\beta_1, \cdots, \beta_N\}$) and eigenvalues ($\{\lambda_1, \cdots, \lambda_N\}$) via the Finite Elements Method (FEM) [11]. Expansion of (S11) in this eigenbasis yields smoothed $\hat{g}_a(\cdot), \hat{g}_\tau(\cdot)$ for choice of parameter k:

$$\hat{g}(y) = \sum_{i=1}^{N} \frac{\langle g, \beta_i \rangle_V \beta_i(y)}{1 - k\lambda_i w(y)} \tag{S12}$$

$$\text{with } \langle g, \beta_i \rangle_V := \sum_{y \in V} \beta_i^*(y) g(y) w(y)$$

Both $\hat{g}_a(\cdot)$ and $\hat{g}_\tau(\cdot)$ are normalized independently so total cross sectional area and numbers of NFTs projected to the surface are conserved before and after smoothing. NFT densities are computed as the ratio of the normalized, smoothed functions: $\frac{\hat{g}_\tau(\cdot)}{\hat{g}_a(\cdot)}$ and plotted over the surfaces of given MTL subregions (see Figure 7).

Supplementary Note 6

Algorithm S.1 Projective LDDMM with In-Plane Transformation

Initialize: $\varphi_0 = \text{Id}$, $\phi_n, n = 1, \ldots, N$

A: Solve for φ:

1. Update and fix ϕ_ns.
2. Solve Projective LDDMM, optimizing (7d, main text) with respect to vector field $v_t, t \in [0, 1]$.
3. Solve for φ_1, integrating O.D.E $\varphi_1 = \int_0^1 v_t \circ \varphi_t dt$.

B: Solve for ϕ_n's:

1. Update and fix φ_1.
2. Optimize (7d, main text) with respect to $\phi_n, n = 1, \ldots, N$.

Return to A

Algorithm S.2 Linear Prediction of Contrast via a Scattering Transform

1. Initialize φ_1, ϕ_n, $n = 1, \ldots, N$.
2. Compute Scattering transform $(S^1_{J_n}(\cdot), \ldots, S^{48}_{J_n}(\cdot))$ for each target $n = 1, \ldots, N$.
3. Compute 6-dimensional PCA basis and projections, $(\psi^1_n, \ldots, \psi^6_n)$ of each scattered image onto corresponding subspace.
4. Compute α_n, $n = 1, \ldots, N$ using the pseudo-inverse (10a, main text) with $\psi^0_n = 1$ for all n.

Algorithm S.3 Projective LDDMM with Crossing Modalities

A: Solve for α_ns using Algorithm S.2

B: Solve for φ:

1. Update and fix ϕ_ns and calculate mean-field predictors (9b, main text) of MRI projection from histology-based PCA basis, $J^\alpha_n = \sum_{k=0}^{6} \alpha^k_n \psi^k_n, n = 1, \ldots, N$.
2. Solve Projective LDDMM, optimizing (7d, main text) with respect to vector field $v_t, t \in [0, 1]$.
3. Solve for φ_1, integrating O.D.E $\varphi_1 = \int_0^1 v_t \circ \varphi_t dt$.

C: Solve for ϕ_ns:

1. Update and fix φ_1.
2. Optimize (7d, main text) with respect to $\phi_n, n = 1, \ldots, N$.

Return to B

Supplementary Tables and Figures

Table S1 10-fold cross validation accuracy statistics for training data of brain sample.

Trial	AUC	Precision	Recall	Accuracy
1	0.9997	0.0455	1.0000	0.9928
2	0.9983	0.0938	0.9917	0.9829
3	0.9963	0.1148	0.9846	0.9706
4	0.9984	0.2079	0.9971	0.9877
5	0.9796	0.3010	0.9242	0.9543
6	0.9915	0.0500	0.9620	0.9597
7	0.9865	0.2462	0.9031	0.9899
8	0.9239	0.0081	0.7079	0.9520
9	0.9989	0.1714	1.000	0.9939
10	0.9867	0.0255	0.9406	0.9454
Average	0.9860	0.1264	0.9411	0.9729

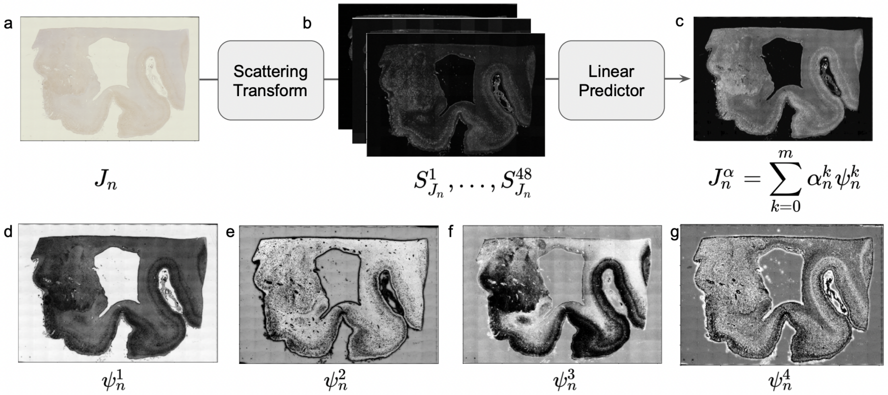

Fig. S1 Algorithm for predicting MR contrast from histology contrast via a Scattering Transform. (a) Initial histology image J_n at 2 μm resolution. (b) Scattering images generated via a Scattering Transform. (c) Output of linear predictor, J^α_n, predicting MRI contrast from Scattering images. (d-g) Projections of scattering images onto first 4 elements of 6-dimensional PCA basis.

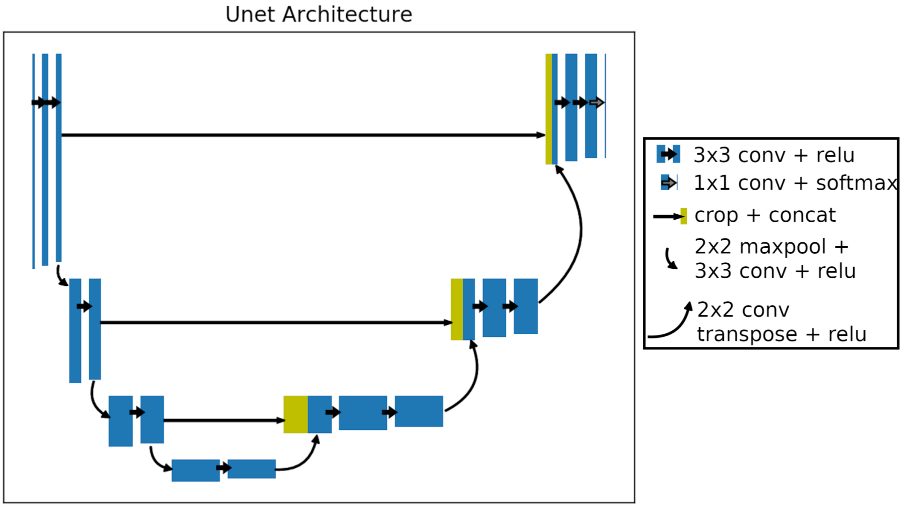

Fig. S2 Structure of UNET trained to detect neurofibrillary tau tangles (NFTs). Input depicted at upper left corner with output at far right. Arrow style reflective of operation (3x3 convolutions with stride 1, rectified linear units, 2x2 max poolings, 2x2 transposed convolutions with stride 2, 1x1 convolution with stride 1, softmax, and concatenation with center cropping). Image size at each stage proportional to size of block: height proportional to number of pixels in image and width proportional to number of channels. Input image size of training data points, here, is 132x132 pixels with 3 channels (RGB).

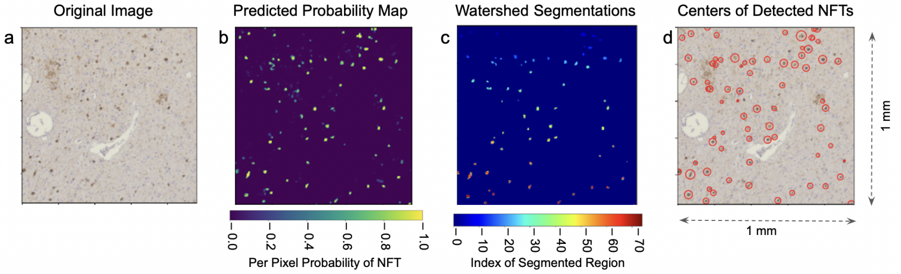

Fig. S3 Output after each step in neurofibrillary tau tangle (NFT) detection algorithm. (a) Input image of histology tissue stained with PHF-1 for tau detection. (b) UNET predicted per-pixel probabilities of belonging to an NFT. (c) Segmentation of connected high probability components following application of the watershed algorithm. Each connected component assigned unique label (2 to 80) and considered a separate NFT. (d) Centers of each NFT shown inscribed in red circle with size proportional to size of detected NFT.

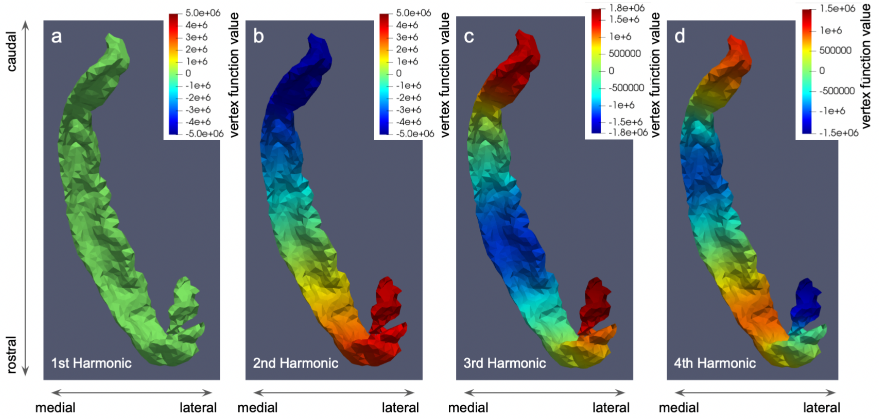

Fig. S4 Basis elements (harmonics) constructed with the Laplace-Beltrami operator for CA1 surface. (a) First harmonic with corresponding eigenvalue of 0. (b) Second harmonic with eigenvalue of -0.0025. (c) Third harmonic with eigenvalue of -0.0081. (d) Fourth harmonic with eigenvalue of -0.0180.

Supplementary References

[1] Snyder, D., Thomas Jr., L., Ter-Pogossian, M.: Mathematical model for positron-emission tomography systems having time-of-flight measurements. IEEE Transactions on Nuclear Science **NS-28**(3), 3575–83 (1981)

[2] Barrett, H.H.: Iii the radon transform and its applications. Progress in Optics, vol. 21, pp. 217–286. Elsevier (1984). https://doi.org/10.1016/S0079-6638(08)70123-9. https://www.sciencedirect.com/science/article/pii/S0079663808701239

[3] Snyder, D., Cox, J.: An overview of reconstruction tomography and limitations imposed by a finite number of projections. In: Proceedings of Workshop on Reconstruction Tomography in Diagnostic Radiology and Nuclear Medicine, Puerto Rico (1975)

[4] Yushkevich, P.A., et al: 3d mapping of tau neurofibrillary tangle pathology in the human medial temporal lobe. In: 2020 IEEE 17th International Symposium on Biomedical Imaging (ISBI), pp. 1312–1316 (2020). https://doi.org/10.1109/ISBI45749.2020.9098462

[5] Mallat, S.: Group invariant scattering. Communications on Pure and Applied Mathematics **65**(10), 1331–1398 (2012)

[6] Bruna, J., Mallat, S.: Invariant scattering convolution networks. IEEE Transactions on Pattern Analysis and Machine Intelligence **35**(8), 1872–1886 (2013) https://arxiv.org/abs/1203.1513. https://doi.org/10.1109/TPAMI.2012.230

[7] Mallat, S.: Recursive interferometric representations. In: European Signal Processing Conference, pp. 716–720 (2010)

[8] Sifre, L., Mallat, S.: Rigid-motion scattering for texture classification [j]. Computer Science **3559**, 501–515 (2014)

[9] Mai, J.K., Paxinos, G., Voss, T.: Atlas of the Human Brain, 3rd edn. Elsevier Inc, New York (2008)

[10] Cheng, S.-W., Dey, T.K., Shewchuk, J., Sahni, S.: Delaunay Mesh Generation. CRC Press Boca Raton, Florida (2013)

[11] Qiu, A., Bitouk, D., Miller, M.I.: Smooth functional and structural maps on the neocortex via orthonormal bases of the laplace-beltrami operator. IEEE Transactions on Medical Imaging **25**(10), 1296–1306 (2006). https://doi.org/10.1109/TMI.2006.882143
